# Long-Term Sequalae of Undiagnosed Intrusion of a Primary Tooth

**DOI:** 10.3390/dj10110202

**Published:** 2022-10-27

**Authors:** Thikrayat Bani-Hani, Rona Leith, Anne C. O’Connell

**Affiliations:** 1 Preventive Department, Faculty of Dentistry, Jordan University of Science and Technology, Irbid 22110, Jordan; 2 Paediatric Dentistry, Trinity College, Dublin Dental University Hospital, D02 F859 Dublin, Ireland

**Keywords:** sequelae, diagnosis, intrusion, primary tooth, hypoplasia

## Abstract

Aims: This case demonstrates the adverse sequelae that can follow a traumatic dental injury at a young age. It also highlights the importance of taking a full history and undertaking a thorough exam, independent of the information in the referral. Case presentation: A 9-year-old boy was referred for treatment of “an extra tooth” and “hypoplastic and non-vital” maxillary left permanent incisors. Examination revealed a sinus tract labial to these incisors (21,22) with increased probing depth. However, the teeth were otherwise normal. The child sustained a fall as a baby and lost one of his primary teeth that was never recovered. Information collected suggested the most likely diagnosis was an undiagnosed complete intrusion of a primary incisor, with subsequent hypoplasia and malalignment in the developing teeth. Management included the removal of the intruded primary tooth and monitoring of the hypoplastic permanent incisors until complete eruption and root maturation. Aesthetic restorations were then provided. The patient was referred for orthodontic correction of the malalignment. Conclusion: Misdiagnosis and inappropriate management of dental trauma can cause additional damage. In this case, endodontic therapy in the permanent incisors was avoided by correct diagnosis. Clinicians have to correctly assess and justify their decisions on each individual case.

## 1. Introduction 

Traumatic dental injuries are common in both primary and permanent dentitions. Recently, it has been estimated that more than one billion living people have had traumatic dental injuries [1]. In children, it was reported that about one-third and one-fourth have sustained dental injuries to their primary and permanent dentitions, respectively [2]. The overall reported incidence of dental injuries in children and adolescents aged 0–19 years was estimated to be 4.5% [3]. Dental trauma prevalence and incidence are generally high worldwide. However, reported figures can vary considerably across countries due to environmental and cultural differences [2], and due to the lack of a standardized classification and recording system for dental injuries [4]. The cause and presentation of dental injuries can also vary widely among different age groups. In the primary dentition, the predominant cause of injuries is falls [3]. This is possibly related to lack of motor coordination in young children and is sometimes due to the child’s inability to evaluate risks [5]. Incisors are the most affected teeth, owing to their most exposed position in the dental arch [2]. Luxation type of injuries are more frequently seen in the primary teeth than fractures, and this is attributed to the resilience and pliability of the alveolar bone in young children [3]. 

Injuries to the primary dentition are often overlooked, mainly due to the perception that they will eventually be replaced by permanent teeth and the limited cooperation for examination and management in young children [5]. Nevertheless, various pulpal and periodontal complications may occur in traumatized teeth [6]. These include crown discoloration, pulp necrosis, pulp canal obliteration, gingival retraction, increased mobility, pathological root resorption, and premature loss of the primary tooth [7]. However, there is a paucity of studies in the literature regarding the time elapsed before the occurrence of sequelae following injuries. In a longitudinal retrospective study, a significant proportion of sequelae occurred within 180 days after dental injuries, however, complications were also diagnosed after longer follow-up periods (more than 4 years) [7]. Moreover, traumatic injuries in the primary dentition can adversely affect the developing permanent teeth due to the close proximity between the apices of the primary teeth and the developing successors. A variety of malformations may occur ranging from mild disturbance in mineralization to a sequestration of the whole tooth germ [8]. The age of the child, the developmental stage of the permanent tooth germ, and the severity and the direction of trauma are all important variables that dictate the extent of tooth damage in developing teeth [9,10]. Intrusive luxations in the primary dentition are considered one of the most serious injuries with a high risk of complications in the traumatized teeth, as well as in the developing successors [10]. 

Careful clinical and radiographic examination of dental injuries along with early diagnosis, timely intervention, and regular follow-up are essential to prevent and minimize complications. In general, the International Association of Dental Traumatology (IADT) guidelines [11] recommend that injuries in the primary dentition are treated conservatively with observational treatment being the most appropriate approach in many situations. Active interventions are mainly required when there is risk of aspiration, ingestion, or interference with the occlusion [11]. For intrusive luxation injuries, watchful waiting for spontaneous re-eruption has been the most recommended treatment modality and has been associated with the least risk of complications [12]. The rationale for this conservative management is to reduce further suffering of the child and to avoid inflicting additional damage to the developing permanent teeth. However, the IADT guidelines [11] recommend that children with significant injuries, such as intrusion or avulsion to their primary teeth, receive checkups until eruption of the permanent teeth for early diagnosis and treatment of possible sequelae [11]. Misdiagnosis and incorrect treatment of dental injuries can further damage the traumatized teeth and lead to unsatisfactory outcomes. This case shows incorrect diagnosis of the original trauma in the primary dentition as well as misinterpretation of its adverse sequalae in the permanent teeth, years later. The current report also highlights the importance of taking a full history and undertaking a thorough examination, independent of the information or diagnosis in the referral. 

## 2. Case Report 

A 9-year-old Nigerian boy was referred by his general dentist for “root canal treatment of hypoplastic and necrotic upper left incisors” and removal of “a supernumerary tooth” in the region. The chief complaint was concern about “discolored and rotated top front teeth with pus discharge in the area”. No history of recent trauma was reported. The child was otherwise physically well and with clear medical background. 

### 2.1. Clinical Examination and Diagnosis 

**Figure 1:**
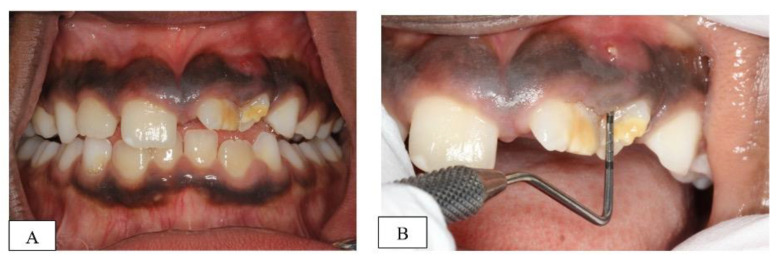
(**A**): frontal clinical view of patient’s dentition at initial assessment, (**B**): closer view of the hypoplastic teeth (21&22) showing the labial sinus tract and the increased probing depth (9 mm).

Clinical examination revealed hypoplastic enamel defects and malalignment of the upper left permanent incisors (teeth # 21&22) with a labial sinus tract between the two teeth (Figure 1A). An increased probing depth, in line with the sinus tract, was also recorded between the two teeth (highest reading 9 mm mid-buccal of 22) (Figure 1B). However, the teeth responded positively to cold sensibility testing with ‘Endo-Frost’ (Roeko; Coltene, Germany), had normal mobility and were not tender to percussion. The child had otherwise intact and caries-free dentition with no previous dental treatment.

**Figure 2:**
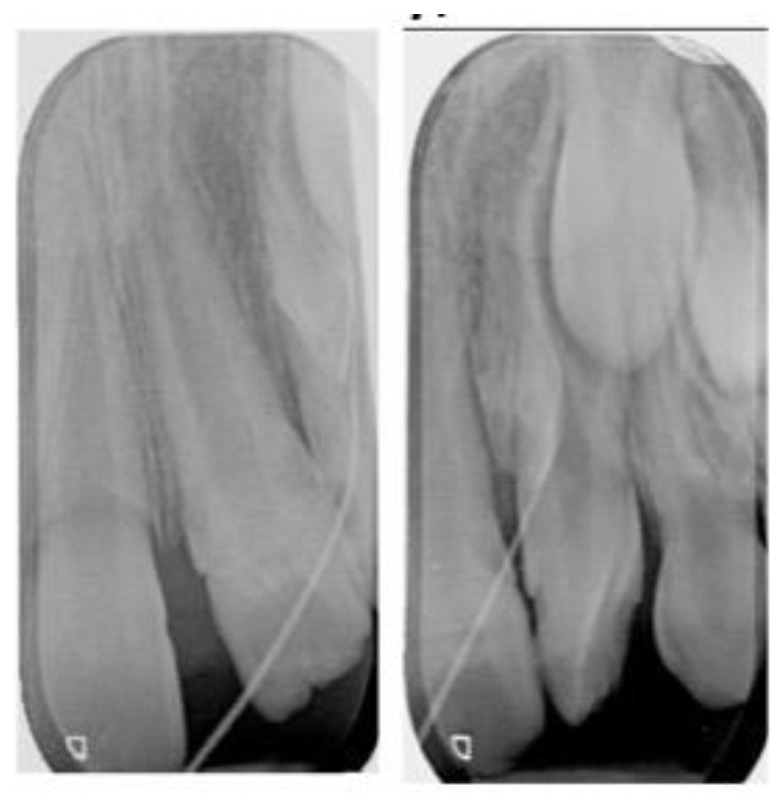
Periapical radiographs at initial assessment taken at different angles to locate the “extra tooth” with a Gutta-Percha point to trace the sinus tract.

Using the horizontal parallax technique, the “extra tooth” appeared to be in a labial position (Figure 2). In the same radiographs, a gutta-percha point was used to trace the origin of the sinus tract. The radiographic evaluation revealed the abscess was related to the “extra tooth” (Figure 2).

Detailed questioning of the child’s parents revealed a traumatic dental injury at the age of 18 months. The mother reported that the child had fallen off his bed at home and “lost” one of his “baby” teeth that was never found at the time of injury. The mother sought assessment at the Accident and Emergency service, however, the missing tooth was erroneously diagnosed as an avulsion by the attending physician. No further intervention was provided. 

A full history and thorough clinical examination, independent of the information in the referral letter, revealed that these permanent incisors were not necrotic and had no sign of pulp degeneration. The ‘extra’ tooth in the radiograph was assumed to be a completely intruded primary incisor and not a supernumerary tooth. 

### 2.2. Treatment Plan and Progress 

**Figure 3:**
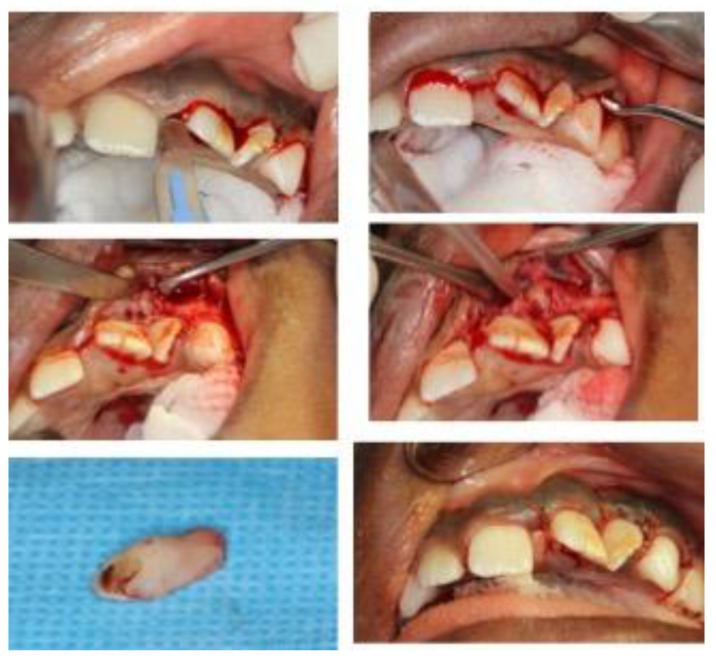
Intra-operative pictures for the surgical removal of the “extra” tooth. Notice the morphology of removed tooth that highly resembles a primary incisor with a mild crown fracture and some apical resorption.

Extraction of the abscessed primary tooth was planned and completed under general anesthesia due to potential behavior management problems and the complicated access to the intruded tooth (Figure 3). Closer inspection of the removed tooth revealed it was a primary incisor and confirmed the preliminary diagnosis. Furthermore, the tooth was located buccal to the cortical plate in a subperiosteal position, and was not incorporated in the growing maxilla. The permanent incisors that had hypoplastic enamel defects were partially erupted at the time of the operation, therefore the decision was made to closely monitor these teeth with intensive prevention until complete eruption. 

**Figure 4:**
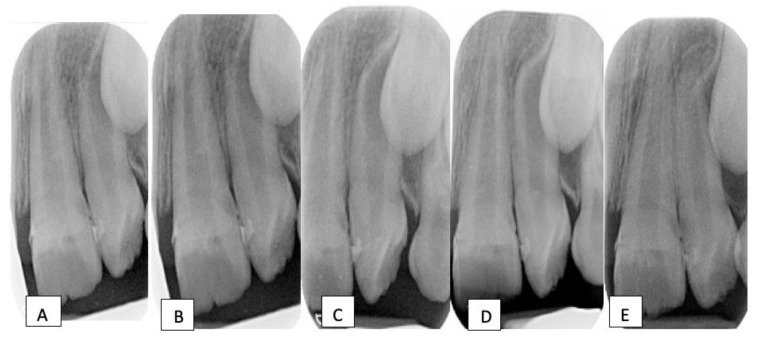
Radiographic evaluation of 11 & 21; (**A**) 2 weeks after the surgery (immediate post-operative review), (**B**) at 3-month, (**C**) at 6-month, (**D**) at 12-month, (**E**) at 24-month recall visits. Notice the continued root development and the apical closure in the hypoplastic teeth.

At the postoperative review (2 weeks after the surgery), there was a satisfactory healing with a complete resolution of the sinus tract. The periodontal examination showed resolution of the increased probing depths. The patient was then placed on a 3-monthly recall interval to monitor the development and eruption of the upper left incisors. Owing to the hypoplastic enamel defects, intermittent sensitivity later developed in the upper left permanent incisors, and desensitizing agents (e.g., tooth mousse) were used and oral hygiene instructions were reinforced. Nevertheless, a normal eruption rate of these incisors with appropriate root formation and satisfactory apical closure was evident on subsequent follow-up visits, confirming pulp vitality and favorable outcomes in these teeth [13] (Figure 4). 

**Figure 5:**
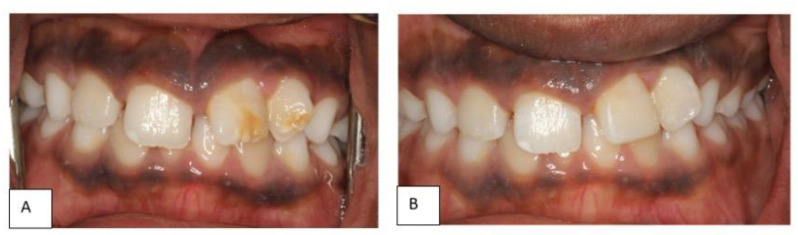
(**A**) the patient’s teeth at a 2-year follow up showing almost complete eruption of upper incisors. (**B**): the hypoplastic teeth after restoration with direct resin composite.

Following complete eruption of the affected permanent incisors, aesthetic restorations were provided with light-cured composite (Z350; 3M ESPE, St. Paul, MN, USA), using a minimally invasive cavity preparation (Figure 5). The aesthetic improvement re-established the patient’s self- esteem, and controlled tooth sensitivity. The patient was then referred for orthodontic treatment for correction of the malalignment. 

## 3. Discussion 

Injuries in the primary dentition, particularly the severe forms, require careful examination and monitoring, not only for the tissue damage and complications in the traumatized primary tooth but also for the possible sequelae in the permanent dentition [14,15,16].

The current IADT guidelines highlight the need to follow severe injuries, especially intrusion and avulsion of primary teeth up until the eruption of the permanent teeth, for early diagnosis and timely management of any complications [11]. Parents should be informed to watch for any unfavorable outcomes and advised to return to the clinic as soon as any unfavorable outcome is identified. It is very important that parents are also informed about any possible damage or complications in the developing permanent teeth. In this case, insufficient information was given to parents at the time of the original trauma, and no follow-on management occurred.

This report represents an unusual case of the complete intrusion of a primary incisor that had failed to re-erupt and remained undiagnosed for years until it developed necrosis and infection. Based on the clinical presentation only, tooth loss (avulsion) was mistakenly assumed in the first place, following the injury. Merkle reported a case of complete intrusion of a primary incisor that was erroneously diagnosed as an avulsion injury by the attending emergency room physician, and later discovered by the dental team during routine care [17]. This illustrated the importance of involving a dental professional in the evaluation of dental trauma cases. The need for radiographic assessment of dental injuries cannot be overemphasized, particularly in situations where teeth appear to be missing to ensure that they are not completely intruded [18].

A report in the literature was located where an intruded primary incisor remained undiagnosed for 15 years [19]. It was accidently discovered on radiographic evaluation for orthodontic treatment; the tooth was initially assumed to be a mesiodens, however, a closer inspection of the structure, following its surgical removal, revealed a deciduous tooth crown which had undergone irregular resorption [19]. In addition, the tooth was easily separated from the cortical plate and left a shallow depression in the surrounding bone. On questioning, the parents recalled the child sustained a fall at 10 months of age and “lost” one of her primary incisors [19]. In the present case report, the permanent incisors had an hypoplastic enamel defect and were malaligned subsequent to the intrusive luxation in the primary tooth. At the age of 18 months (when the injury occurred), the permanent incisors are expected to be at the crown formation and calcification stage. It is likely that the path of intrusion had injured and displaced the developing teeth germs (# 21&22), resulting in hypoplasia and rotation. In fact, intrusive luxation bears the highest risk of damaging the permanent tooth germ. Lauridsen et al. suggested the damage may be due to direct mechanical injury caused by the displaced primary tooth or by long-term complications such as pulp necrosis and infection with periapical inflammation or impaction of the primary tooth [6]. In the present case, the two likely causes of damage exist. The displacement and rotation of the teeth is a likely consequence of deflection around the intruded tooth.

It is worth mentioning that the provisional diagnosis that was made by the referring dentist (the presence of a supernumerary tooth rather than a completely intruded tooth) was included in the differential diagnoses and could not be initially ruled out. However, the trauma history (i.e., loss of a baby tooth that was never found at the time of injury) and the quite localized enamel defects of the developing incisors in the area, in addition to their rotation in an otherwise perfectly aligned dentition, all increased the suspicion of dental trauma. That said, this preliminary diagnosis was not proved until the tooth was surgically removed and inspected closely to confirm the morphology of a primary incisor. Furthermore, the tooth was located buccal to the cortical plate in a subperiosteal position and was not incorporated in the growing maxilla.

## 4. Conclusions and Clinical Implications

Dental injuries in young children can have a significant negative impact on oral health with esthetic, functional, and psychological consequences. Careful clinical and radiographic examination, along with regular follow-up, is essential in all traumatic injuries. Organization of a proper emergency service and first aid for dental trauma is paramount at all pediatric hospitals, with the presence of a dental professional at initial assessment. Onward referral to a dentist after the acute emergency should be an essential component in the management of traumatic dental injuries. Inaccurate or incomplete diagnosis and subsequent inappropriate treatment protocol can cause additional damage and an unsatisfactory outcome. Clinicians must always make their own decision on each individual case, independent of the information or preliminary diagnosis from the referring dentist.
